# Effectiveness of Pfizer/BioNTech and Sinopharm COVID-19 vaccines in reducing hospital admissions in prince Hamza hospital, Jordan

**DOI:** 10.3389/fpubh.2022.1008521

**Published:** 2022-09-21

**Authors:** Hafez Al-Momani, Khawla Aldajah, Ebtisam Alda'ajah, Yousef ALjafar, Zainab Abushawer

**Affiliations:** ^1^Basic Medical Science Department, School of Medicine, Hashemite University, Zarqa, Jordan; ^2^Infection Control Unit, Prince Hamza Hospital, Amman, Jordan

**Keywords:** COVID-19, hospital admission, Pfizer, Sinopharm, Jordan

## Abstract

**Background:**

There is a need to establish the effectiveness of the coronavirus disease 2019 (COVID-19) vaccines in reducing COVID-19-related hopitalization of patients in Jordan. As the vaccination program accelerates, it is important to determine whether the vaccines' effectiveness (VE) has successfully reduced the number of acute cases admitted to hospital.

**Methods:**

To determine the efficacy of Pfizer-BioNTech and Sinopharm COVID-19 vaccines among Jordanian patients admitted to Prince Hamza hospital, a single center case-control study was performed. The study analyzed the hospitalization rates of vaccinated (*n* = 536) and unvaccinated (*n* = 585) individuals across the 2-month period from February 6 to April 6, 2022. The cases were patients who tested positive for SARS-CoV-2 (“case-patients”), whilst the control group were hospital patients who did not test positive for SARS-CoV-2 (“control-patients”).

**Results:**

This study found that among 1,121 total participants (561 cases and 560 control), the overall vaccine effectiveness (VE) among the participants was 84% (95% Cl 79–88%). VE was higher in females (88%, 95% Cl 84–93%) than in males (77%, 95% Cl 67–84%) (*p* < 0.001), and it was highest in those between the ages of 18 and 28-years-old (95%, 95% CI 86–98%). For patients with pre-existing conditions, including chronic heart disease, chronic lung disease, and diabetes, VE was higher compared to patients with no comorbidities, though the difference was not statistically significant. Finally, in comparing all vaccinated participants, VE was higher for those who received the Pfizer vaccine (VE = 92%, 95% CI 88–94%) (OR 0.08, 95% CI 0.06–0.12) than for those who received the Sinopharm vaccine (VE = 67%, 95% CI 52–78%) (OR 0.33, 95% CI 0.22–0.48); (*p* = 0.011).

**Conclusion:**

Overall, Pfizer and Sinopharm vaccines were found to be effective in limiting hospitalizations for acute cases of coronavirus among Jordanian adult's patient's cohort between February 6 and April 6, 2022, especially among patients with comorbidities.

## Introduction

In December 2019, the acute respiratory syndrome coronavirus (SARS-CoV-2) emerged in China and rapidly spread across the world, jumpstarting a global pandemic that has persisted (1). As scientists and medical professionals all over the world have turned their attention to fighting COVID-19, a multitude of medications have been proposed with therapeutic capability, including Camostat, Darunavir, Ivermectin, Remdesivir, Resveratrol, and Ritonavir (2, 3). Moreover, a considerable efforts are being made globally to develop safe and effective vaccines against coronavirus as a primary prophylactic intervention.

Many companies have introduced candidate vaccines, each with various indications, contraindications, and adverse events, but ultimately all providing differing levels of efficacy in preventing infection, acute outcomes, and death as a result of coronavirus infection (4). Therefore, evaluating the effectiveness of authorized vaccinations is vital. At the time that the data for this study was collected, Jordan was undergoing its third wave of coronavirus, which has been attributed to the highly transmittable Omicron variant. As of March 23, 2022, the number of positive cases was recorded to be 1,689,314, and there were 14,003 deaths, and the situation has only escalated since (5). For a country with approximately 10 million people, this rate of infection and death toll represents a significant portion of the population. The government has consequently enforced stricter safety measures to combat the outbreak (6). Furthermore, while the Ministry of Health in Jordan launched a national vaccination campaign on December 23, 2020, inviting everyone who lives in the country to register for free coronavirus vaccinations, only about 4.41 million (~43.2%) of the population have been fully vaccinated at this point.

One of the several studies that have considered explanations for the country's low vaccination population found that misinformation and conspiracy theories, primarily ones that discredit the vaccine's ability to reduce rates of hospitalization, have had a negative impact on vaccine administration among Jordanians (7). As this may be a major impediment in Jordanians' willingness to receive the vaccination, determining various vaccines' effectiveness in limiting rates of hospitalization due to acute cases of coronavirus is paramount for the country's overall competency in managing the pandemic. Therefore, this study will compare the most commonly administered vaccines among Jordanian adults admitted to one governmental hospital in Jordan—the mRNA Pfizer-BioNTech vaccine and the inactive Sinopharm vaccine—to determine their overall efficacy in limiting hospitalization.

## Methodology

### Study design

To consider each vaccine's effectiveness in preventing coronavirus-related hospitalizations among sample of Jordanians patients, this study used a retrospective case-controlled analysis of 1,121 adults over the age of 18 years who were hospitalized at Prince Hamza Hospital in Jordan between February 6 and April 6, 2022. Prince Hamzah Hospital is the main isolation and treatment center for COVID-19 in Jordan. The sample consisted of patients who tested positive for SARS-CoV-2 and had also received either the Pfizer-BioNTech or the Sinopharm vaccination. The control group was comprised of patients who were admitted to the hospital but did not test positive for SARS-CoV-2. Any individuals with immune compromising conditions were excluded from the pool.

The pool of hospitalized patients due to coronavirus consisted of individuals who had both a positive test for SARS-CoV-2 within 10 days of symptom onset and a diagnosis of a clinical syndrome that signals an acute case of coronavirus, which includes ≥1 of the following: fever, cough, shortness of breath, loss of smell, requiring respiratory support, or new pulmonary findings on chest imaging consistent with pneumonia. The control group consisted of patients who were hospitalized without an indication of acute coronavirus and who tested negative for SARS-CoV-2.

### Data collection

A standardized medical record review provided demographic information including age, gender, medical history, SARS-CoV-2 vaccination status, and other patient characteristics. Specific details of patients' SARS-CoV-2 vaccine administrations, including dates and vaccine suppliers, were supplied through source verification of documents like vaccine cards or hospital records.

### Classification of vaccination status

Patients' vaccination status was categorized based on the number of vaccine doses received before the reference date (i.e., the date of symptom onset for coronavirus-positive patients), (“case-patients”), and the date of hospitalization for coronavirus-negative patients (“control-patients”). All hospitalized patients were determined to be either “fully vaccinated” or “unvaccinated.” Because both Pfizer and Sinopharm SARS-CoV-2 vaccinations were administered as a two-dose series and protective immunity is not expected immediately after the first dose, participants were only considered “fully vaccinated” fourteen days after receipt of the second vaccine dose (8). Subsequently, patients who had received no vaccine before the reference date were considered “unvaccinated.” All other vaccine scenarios, including those who received the first dose less than fourteen days before the reference date were excluded from the study. This included patients who received vaccinations from vaccine suppliers other than Pfizer-BioNTech or Sinopharm, vaccines that were not authorized in Jordan, patients who received vaccine doses from different suppliers, or patients who only received one dose. Patients who had previously contracted coronavirus were also excluded from the study.

### Statistical analysis

By comparing the vaccination status of case patients and control patients, VE was calculated using the following expression: VE = (1–odds ratio) × 100% (9). The 95% confidence intervals (CI) were determined using the formula 1–CI_OR_, where CI_OR_ is the confidence interval of the odds ratio estimates.

VE estimates were stratified by age group, designated in 10-year increments (18–28, 28–38, 38–48, 58–68, and >68-years-old), SARS-CoV-2 vaccine supplier (Pfizer-BioNTech or Sinopharm), and the following underlying medical conditions: diabetes mellitus, chronic lung disease, chronic cardiovascular disease, and obesity. Characteristics of cases and controls were compared by employing chi-square tests or Fisher's exact tests for categorical variables and Student's *t*-test or Wilcoxon rank-sum tests for continuous variables.

Hashemite University and Prince Hamza Hospital's Ethics Service Committee granted ethical approval for this case study (reference number 5/3/2020/2021).

## Results

**Figure 1:**
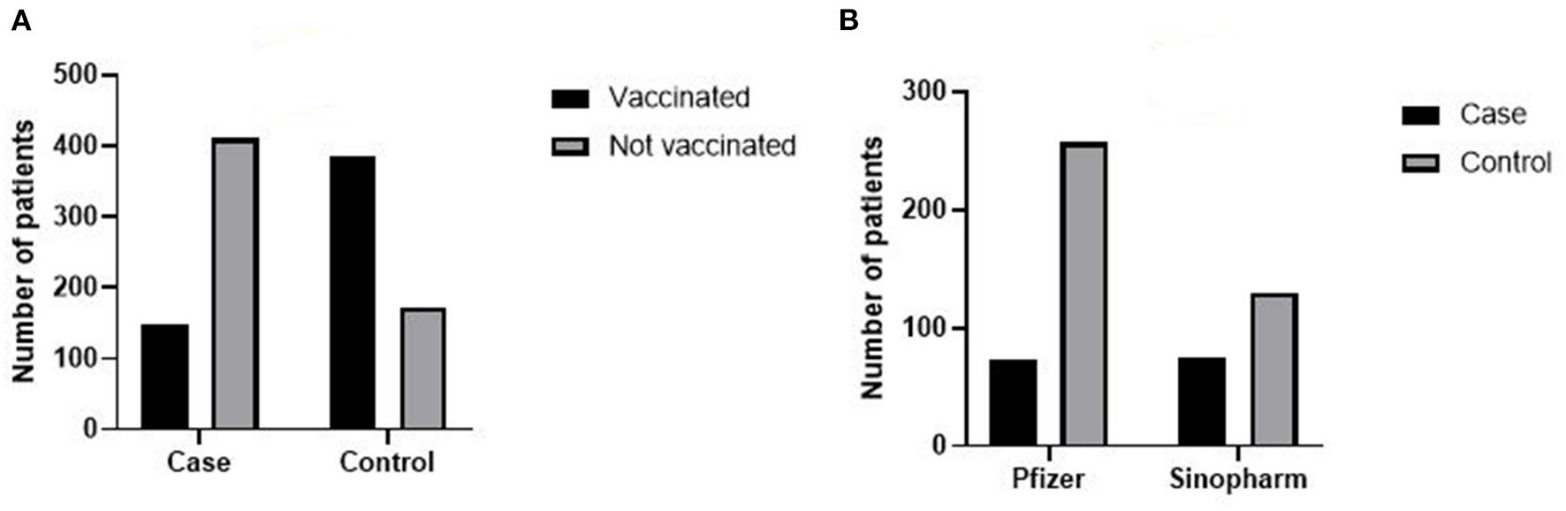
**(A)** Number of vaccinated and non-vaccinated patient among cases and control group case and control patient included in this study. **(B)** Number of patient vaccinated with Pfizer and Sinopharm COVID-19 vaccination among cases and control.

A total of 186 patients, who were hospitalized at Prince Hamza Hospital between February 6 and April 6, 2022, were excluded from this study. Of these, 36 had an immune compromising condition, 66 had received ≥1 vaccine dose other than a Pfizer-BioNTech or Sinopharm vaccine, and 84 did not meet other eligibility criteria. The remaining 1,121 recorded patients included 561 case-patients and 560 control-patients. Overall, 585 (52%) patients were unvaccinated and 536 (47.5%) were vaccinated. Of those who were vaccinated, 205 (18%) were fully vaccinated with the Sinopharm vaccine and 331 (29.5%) were fully vaccinated with the Pfizer-BioNTech vaccine (Figure 1). Demographically, 51.4% of all participants were female while the remaining 48.6 % were male. The median age of the participants was 58-years-old. While most cases occurred in individuals between the ages of 38 and 68-years, 22.6% of recorded cases were individuals below the age of 38-years and 14% of cases were individuals over the age of 68 (Table 1). The age distribution among vaccinated and non-vaccinated groups aligned approximately equally, revealing an appropriate parallel from which to draw accurate conclusions between the control and test groups.

**Table 1:** Characteristics of Jordanian hospitalized COVID-19 case-patients and controls.

**Characteristic**	**Cases** ** (*n =* 561)**	**Control** ** (*n =* 560)**	***P***
**Age group**			
18–28	43	50	0.32
28–38	84	78	0.07
38–48	101	93	0.1
48–58	148	134	0.24
58–68	106	119	0.49
>68	79	86	0.014
**Gender**			
Male	256	288	0.97
Female	305	272	0.91
**Underlying medical conditions**			
Chronic cardiovascular disease	123	117	0.23
Chronic lung disease	17	19	0.29
Diabetes mellitus	153	120	0.37

The medical record review concluded that 52% of case-patients and 45.7% of control-patients had at least one underlying condition (*p* > 0.001). The most prevalent underlying conditions reported for both case-patients and control-patients, respectively, were chronic heart disease (22%; 21%) and diabetes (27%; 21.4%) (Table 1). Finally, the median time between the final vaccine dose and symptom onset was 23 weeks for case-patients (IQR 15.3, 32.5) and 23.5 weeks for control-patients (IQR 14.7, 32.1).

The risk of coronavirus infection among vaccinated groups was 0.28 (149/536) while the risk of coronavirus infection among unvaccinated groups was 0.7 (418/585), suggesting a risk ration of 0.4 and odd ration of 0.16 (95% CI 0.12–0.21). VE among hospitalized patients included in the sample was 84% (95 CI 79–88%). In terms of VE by supplier, those who had received the Pfizer-BioNTech vaccine had a higher VE rate (VE = 92%, 95% CI 88–94%) (OR 0.08, 0.06–0.12) than the Sinopharm vaccine (VE = 67%, 95% CI 52–78%) (OR 0.33, 95% CI 0.22–0.48) (*p* = 0.011). Additionally, the risk of coronavirus infection among patients who received the Pfizer-BioNTech was 0.22 compared to 0.36 among those who received the Sinopharm vaccination.

Demographically, point estimates were higher for people ages 18–28-years-old (95%; 95% CI 86–98%) than any other age range. For those with comorbidities, VE was higher for patients with underlying cardiovascular disease (90.0%; 95% CI 83–94%), chronic lung disease (92%; 95% CI 84–96%), and diabetes mellitus (88%; 95% CI 81–93%) compared with patients who had no underlying conditions (Table 2).

**Table 2:** Vaccine effectiveness of Pfizer-BioNTech and Sinopharm vaccines against COVID-19 hospitalization overall and by subgroup.

**Subgroup**	**Vaccinated cases**	**Vaccinated control**	**Odds ratio (95% CI)**	**Vaccine effectiveness (95% CI)**	
	**Patient/total case patient (%)**	**Patient/total control patient (%)**			
**Overall**	149/561 (27.7)	387/560 (72.3%)	0.16 (0.12–0.21)	84% (79–88%)	*P < *0.0001
18–28	6/45 (13%)	38/50 (76%)	0.05 (0.02–0.14)	95% (86–98%)	*P < *0.0001
28–38	23/84 (27%)	58/81 (71.6%)	0.15 (0.08–0.30)	85% (70–92%)	*P < *0.0001
38–48	24/101 (23.7%)	63/90 (70%)	0.13 (0.07–0.25)	87% (75–93%)	*P < *0.0001
48–58	52/141 (36.8%)	89/130 (968.4%)	0.27 (0.16–0.45)	73% (55–84%)	*P < *0.0001
58–68	27/112 (24%)	78/121 (64.5%)	0.185 (0.10–0.31)	81% (69–90%)	*P < *0.0001
>68	17/78 (21.8%)	61/88 (69%)	0.12 (0.06–0.25)	88% (75–94%)	*P < *0.0001
Sinopharm	75/205 (36.5%)	130/205 (63.5%)	0.33(0.22–0.48)	67% (52%−78%)	*P < *0.0001
Pfizer	74/331 (22.3%)	257/331 (77.7%)	0.08 (0.06–0.12)	92% (88–94%)	*P < *0.0001
**Chronic cardiovascular disease**	51/200 (14%)	113/146	0.10 (0.06–0.17)	90% (83–94%)	*P < *0.0001
No chronic cardiovascular disease	98/361	274/414	0.19 (0.14–0.26)	81% (74–86%)	*P < *0.0001
Chronic lung disease	27/108	86/107	0.08 (0.04–0.16)	92% (84–96%)	*P < *0.0001
No chronic lung disease	122/453	301/453	0.17 (0.14–0.28)	83% (72–86%)	*P < *0.0001
Diabetes mellitus	56/205	103/135	0.12 (0.07–0.19)	88% (81–93%)	*P < *0.0001
No Diabetes mellitus	93/356	284/407	0.15 (0.11–0.21)	85% (81–89%)	*P < *0.0001
Obesity by body mass index	30/113	74/107	0.16 (0.09–0.29)	84% (71–91%)	*P < *0.0001
No obesity	119/448	313/453	0.16 (0.12–0.22)	84% (82–88%)	*P < *0.0001
**Gender**					
Male	71/256 (27.7%)	180/288 (62.5%)	0.23 (0.16–0.33)	77% (67–84%)	*P < *0.0001
Female	78/305 (25.5%)	207/272 (76%)	0.12 (0.07–0.16)	88% (84–93%)	*P < *0.0001

**Figure 2:**
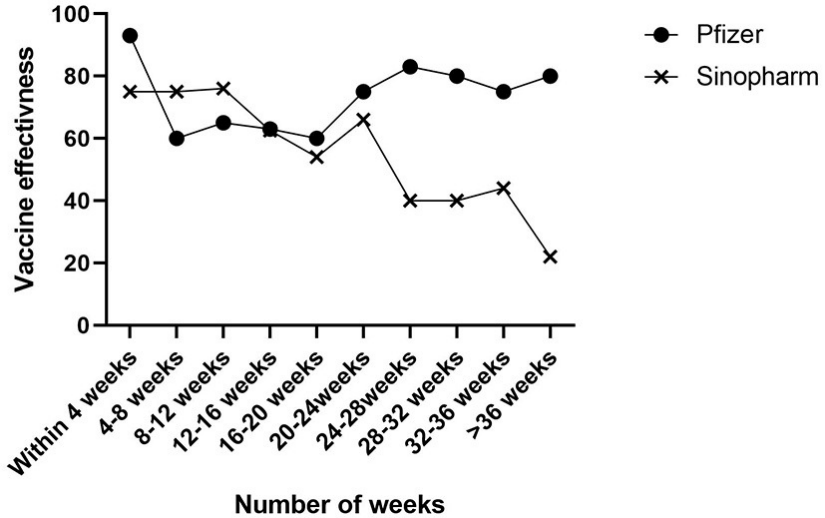
Change in vaccine effectiveness based on number of week after 2 dose Pfizer and Sinpoharm COVID-19 vaccination.

While the Pfizer-BioNTech vaccine's effectiveness against infection increased only after the first twenty weeks following the vaccination, the Sinopharm vaccine markedly decreased 4 weeks after the final dose was administered (Figure 2).

## Discussion

An essential element of managing the COVID-19 pandemic involves vaccines. Our study indicates that two doses of Pfizer-BioNTech or Sinopharm vaccines at least fourteen days after administration provided significant reduction in coronavirus-associated hospitalizations at Prince Hamza Hospital in Jordan. Based upon the hospitalization rates of patients in our patient cohort, it is evident that Sinopharm (VE 92%) is less effective than Pfizer-BioNTech (VE 67%) These results are consistent with previous studies, in particular, those done during the period between March and July, and February and August 2021, which showed the VE of Pfizer-BioNTech and Sinopharm against hospitalization rates to be 86% (95% CI = 82–88%) (10, 11), and 79.6% (95% CI 77.7–81.3%) (12).

On the other hand, this study finds that the VE is lower than in some other assessments in preventing hospitalization due to coronavirus. These other case studies, which examined the same vaccination suppliers as this study, suggested that the VE for fully vaccinated individuals to be 96% (95% CI 49–99%) among adults who received Pfizer-BioNTech vaccines and 81% (95% CI 88–93%) among adults who received the Sinopharm vaccines (13, 14). This study of a sample of hospitalized Jordanians occurred between February 7 and April 7, 2022, when the dominant SARS-CoV-2 variant was considered to be Omicron. This variant surpassed the former Delta variant as the dominant circulating virus in Jordan early in January 2022. Several studies targeted the Delta variant specifically, possibly accounting for the observed differences in VE values among other studies. For that reason, it is critical to interpret VE results cautiously and draw careful comparisons to other vaccine-effectiveness studies conducted in other contexts.

The findings of this study revealed that although both vaccines alleviated the risk of being hospitalized, each offered a different level of protection. For instance, patients given the Pfizer-BioNTech formula exhibited comparatively modest attenuation of VE, and they were more protected than their counterparts, whom received the Sinopharm vaccine, but their protection levels fell significantly. Furthermore, it supports other emerging evidence to suggest that, while VE waning is an expected result following the second dose of both Pfizer-BioNTech and Sinopharm vaccines (15–17), the Sinopharm vaccine in particular is less effective against the Omicron variant, as its initial effectiveness steadily declines 1 month following the second dose (18).

Our findings can be contextualized against several other studies vaccines' efficiency levels. For instance, an assessment of registries in Sweden indicated a sharp fall in vaccine efficacy against the risk of being hospitalized after 25 weeks (19). Meanwhile, Feikin, Higdon (20) conducted a systematic evaluation, which determined that in the 6-month post-vaccination period, vaccine efficacy against the acute risk of disease fell by 9.7 percentage points (95% CI 5.9–14.7). According to Mateo-Urdiales, Alegiani (21), the fall in vaccine efficacy became less evident at the 6-month mark, although researchers found a hint of a possible decrease in relation to the rising Delta variant cases at the conclusion of the follow-up phase.

Echoing the findings of Niessen et al. (22), we found higher VE in patients with comorbidities than in patients without comorbidities. In Niessen and Knol's study, a subgroup analysis of various comorbid conditions found partial and full vaccination of COVID-19 patients conferred some protection for all the comorbidities evaluated. Excluding immune compromised patients, the estimated VE for full vaccination exceeded 96% (95% CI 77–99) for comorbid patients, whereas the VE in patients without comorbidities was 93% (95% CI 82–98) (22). However, these results contradict other research, which found reduced VE in diabetic patients (23), and those with more than one comorbidity (24). For example, Yelin et al. (25) found a negative association between VE and the chronic comorbidities of COPD, immunosuppression, hypertension and type 2 diabetes. However, according to Pellini et al. (28), the efficacy and safety of COVID-19 vaccines in comorbid patients are comparable to that of non-comorbid patients. One explanation proposed by Godbout and Drolet (29) that could account for our findings is that comorbid patients had less social interaction than patients without underlying conditions had. The difference in the number of contacts between the two populations prior to the Christmas 2020/2021 holidays was statistically significant (comorbid contacts = 2.9 (95% CI 2.5–3.2) vs. non-comorbid contacts = 3.9 (95% CI 3.5–4.3); *P* < 0.001) (26).

Our study found that the maximum VE occurred in patients aged 18–28-years-old (95%; 95% CI 86–98%). This finding is similar to those reported by other researchers, who found the antibody response to be greater in younger people than in older people (27–29). However, these finding are not unanimous, as Salmerón Ríos, Mas Romero (30) did not detect any relationship between antibody response and age, though their findings could be limited by its sample, which was of residents in long-term care facilities. The participants in that study ranged in age from 65 to 99-years-old (mean 82.9 years) and by virtue of being in long-term care, had various disabilities and frailties. The majority of the studies found that a subset of pro-inflammatory B cells increased and the quality of memory B cells and plasma cells was affected, which resulted in a reduced humoral immune response (28). Furthermore, the rate of change in titers of antibodies in people < 50-years-old were appreciably lower than those of people older than fifty (29). Although there was a marked difference in the antibody response after first dose of COVID-19 vaccine, the response diminished over time; this effect was more pronounced following the second dose (29, 31). The vaccine-initiated antibody response has implications for COVID-19 vaccination programs, indicating that to maintain the response in older people, multiple boosters are required (32). These findings also emphasize the benefits of implementing strategies and individualized vaccination programs that can minimize the age-related inadequacies of the COVID-19 vaccines (33).

In a comparison of the sexes, we found VE was greater in females than males; this observation may be attributed to hormonal differences between the sexes. It is recognized that estradiol in females promotes adaptive and innate immune responses, whereas these same responses are dulled by testosterone in males; therefore, the antibody response is greater in females than males (34). Notarte, Ver (35) also noted that the humoral response and adverse events due to the COVID-19 mRNA vaccines is greater in females.

An important finding reported by Ma, Hao (36) is that replication of the SARS-CoV-2 virus can be inhibited directly by estrogen. The hormone limits the incidence of SARS-CoV-2 infection modifying cell metabolism genes, thereby sustaining cell integrity and enhancing metabolic function. Conversely, immune cell activity and androgen receptors are subdued by testosterone, which reduces inflammation and stimulates anti-inflammatory responses. Consequently, compared to males, females have an innate physiologic lead when initiating immune responses to infections (36).

Limitations of this study include diversity of the sample pool, identification of the virus variant, and antibody measurements. First, this study did not consider children, immune compromised adults, or individuals who tested positive for coronavirus but were not hospitalized. Second, supplier-specific effectiveness among a variety of virus variants could not be determined as variants were largely unknown. Thirdly, our study is disadvantaged by inconsistent serological undertakings at admission. This means we could not assess immune status prior to hospitalization; nor could we quantify vaccine-induced antibody levels to correlate with vaccine effectiveness. This information would have enabled us to develop deeper and broader knowledge about the effectiveness of the vaccines.

Further, estimates of vaccine effectiveness could be compounded by certain behavioral measures that were not considered in this study. For example, the use of non-pharmaceutical interventions, including mask use, social distancing, and exposure risks have been found to be useful in preventing coronavirus infection, much apart from one's vaccination status (37).

In conclusion, this study demonstrated that Pfizer-BioNTech and Sinopharm vaccines were effective in reducing the rate of hospitalization among a sample of 1,121 adult Jordanians patients between February 7 and April 7, 2022. Vaccines were found to be particularly effective for patients with comorbidities and younger age groups. In addition, this research emphasizes the importance of monitoring vaccine effectiveness over time, rather than at an isolated moment. It reiterates the useful and increasingly relevant role served by booster doses in restoring high levels of protection that were observed early in the vaccination roll out. Understanding vaccine effectiveness by vaccine supplier can guide individual choices and policy recommendations regarding the continued administration of coronavirus vaccines, as well as subsequent boosters in providing substantial and significant protection against coronavirus hospitalization. Moving forward, this study hopes to add to the ongoing research and increasing information around preventative measures to fight coronavirus. Future research that explores the interdependence of age, comorbidities, serostatus, and sex and the relationships between them with humoral responses is warranted. Also, studies could compare the extent and nature of humoral responses of other COVID-19 vaccines, such as Johnson & Johnson, and Moderna (mRNA 1273), and the vaccines evaluated in this study.

## Data availability statement

The original contributions presented in the study are included in the article/Supplementary material, further inquiries can be directed to the corresponding author.

## Ethics statement

The studies involving human participants were reviewed and approved by Hashemite University and Prince Hamza Hospital's Ethics Service Committee (reference number 5/3/2020/2021). The patients/participants provided their written informed consent to participate in this study.

## Author contributions

HA-M was responsible for the study design, analyzed data, and wrote the manuscript. KA, EA, YA, and ZA responsible for data collection and co-wrote the manuscript. All authors contributed to the article and approved the submitted version.

## Conflict of interest

The authors declare that the research was conducted in the absence of any commercial or financial relationships that could be construed as a potential conflict of interest.

## Publisher's note

All claims expressed in this article are solely those of the authors and do not necessarily represent those of their affiliated organizations, or those of the publisher, the editors and the reviewers. Any product that may be evaluated in this article, or claim that may be made by its manufacturer, is not guaranteed or endorsed by the publisher.
