# Supplementary material for: Effectiveness of Pfizer/BioNTech and Sinopharm COVID-19 vaccines in reducing hospital admissions in prince Hamza hospital, Jordan
Source: Front Public Health. 2022 Sep 21;10:1008521. doi: 10.3389/fpubh.2022.1008521 (PMC9532618; doi:10.3389/fpubh.2022.1008521)
Supplement: Supplementary file 1 [file Table_1.docx]

**Supplementary Material**

Supplementary Table 1: Number of weeks from the second dose till patients admition to the hosptial

|  | **Cases (n=561)** | | **control (n=560)** | |
| --- | --- | --- | --- | --- |
|  | Sinopharm (n=75) | Pfizer (n=74) | Sinopharm (n=130 ) | Pfizer (n=257) |
| Within 4 weeks | 2 | 0 | 8 | 6 |
| 4-8 weeks | 4 | 7 | 16 | 5 |
| 8-12 weeks | 5 | 5 | 21 | 14 |
| 12-16 weeks | 6 | 11 | 16 | 23 |
| 16-20 weeks | 7 | 11 | 15 | 27 |
| 20-24weeks | 8 | 10 | 10 | 39 |
| 24-28weeks | 11 | 6 | 7 | 34 |
| 28-32 weeks | 9 | 7 | 15 | 33 |
| 32-36 weeks | 12 | 9 | 13 | 36 |
| >36 weeks | 11 | 8 | 9 | 40 |
